# ZNF197-AS1/miR-425/GABARAPL1 axis: a novel regulatory mechanism in uveal melanoma

**DOI:** 10.1152/ajpcell.00457.2024

**Published:** 2024-09-23

**Authors:** Chao Zhang, Shuai Wu

**Affiliations:** ^1^Department of Strabismus and Pediatric Ophthalmology, The Second Hospital of Jilin University, Changchun, People’s Republic of China; ^2^Department of Orbital Disease and Ocular Plastic Surgery, The Second Hospital of Jilin University, Changchun, People’s Republic of China

**Keywords:** ceRNA regulatory network, GABARAPL1, miR-425, uveal melanoma, *ZNF197-AS1*

## Abstract

This study investigates the role of the long noncoding RNA (lncRNA) *ZNF197-AS1* in uveal melanoma (UM), focusing on its function within a competing endogenous RNA (ceRNA) network. Using the UM-related TCGA (The Cancer Genome Atlas) dataset, we analyzed the expression levels of *ZNF197-AS1* and its correlation with *miR-425* and *GABARAPL1*, an essential autophagy-related gene. Our analysis revealed that *ZNF197-AS1* acts as a ceRNA by competitively binding to *miR-425*, resulting in the upregulation of *GABARAPL1*. This interaction plays a crucial role in the growth and metastasis of UM. The expression of *GABARAPL1* showed a strong correlation with the clinical outcomes of patients with UM. Furthermore, in vitro assays confirmed that *ZNF197-AS1* impedes UM cell proliferation, migration, and invasion by modulating the *miR-425/GABARAPL1* axis. These findings suggest that ZNF197-AS1 can effectively inhibit UM progression through this ceRNA regulatory network. This study provides valuable insights into the molecular mechanisms underlying UM and highlights the potential of targeting the *ZNF197-AS1/miR-425/GABARAPL1* axis as a therapeutic strategy for UM.

**NEW & NOTEWORTHY** This study identifies the ZNF197-AS1/miR-425/GABARAPL1 axis as a novel regulatory mechanism in uveal melanoma. ZNF197-AS1 upregulates GABARAPL1 by sponging miR-425, inhibiting UM cell proliferation, migration, and invasion. This discovery highlights a potential therapeutic target, providing new insights into UM progression and patient outcomes.

## Graphical abstract

**Figure:**
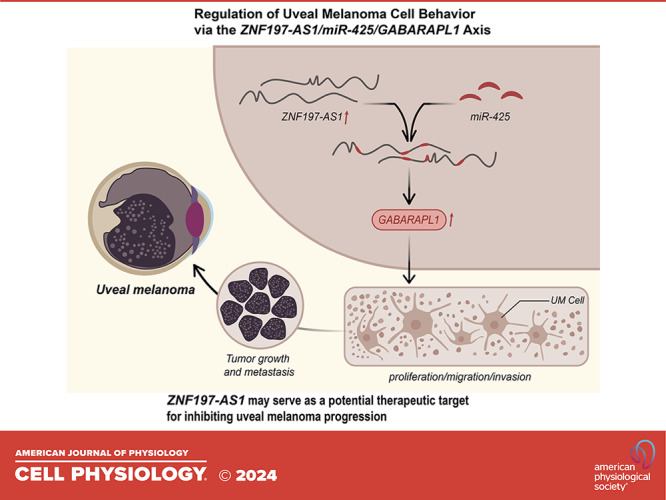

## INTRODUCTION

Uveal melanoma (UM) is an aggressive cancer from melanocytes in the eye; ∼50% of all patients develop metastatic disease, even with the successful treatment of primary tumors with radiation or surgery (1). In addition, metastases tend to disseminate to the liver, and the prognosis of patients with metastatic UM remains poor (2, 3). Therefore, a better understanding of the molecular factors in the metastases and the underlying mechanism can provide additional treatment approaches for UM.

According to the recent classification by the World Health Organization, melanoma is further subdivided into those associated with sunlight exposure and those unrelated. The former can lead to skin differentiation into melanomas of low and high sun damage levels based on chronic sun damage (CSD) (4). The etiology primarily unrelated to sunlight exposure melanomas includes mucosal, acral, uveal, and spitzoid melanomas; blue or congenital nevi; and rare melanomas arising in the central nervous system (5–7). Despite UVB radiation being a recognized environmental carcinogen, it is vital for the skin to produce vitamin D3, which can be enzymatically converted into biologically active hydroxyl derivatives. These compounds exhibit multifunctional effects, including anti-melanoma activity and protection or repair of oxidative stress and DNA damage induced by solar radiation. Furthermore, mounting evidence suggests that vitamin D deficiency, defined as ≤20 ng/mL (50 nmol/L) 25(OH)D3, and vitamin D signaling defects involving vitamin D receptor and CYP27B1 pathways impact the probability and natural history of melanoma development, including overall survival (OS), disease-free survival, and treatment response (8).

Long noncoding RNAs (lncRNAs) have been promising targets in personalized medicine and serve as potential biomarkers for many diseases, including UM, due to their crucial roles in proliferation, invasion, apoptosis evasion, and drug resistance by affecting oncogenes and tumor suppressor genes (9). A recent study has identified *ZNF197-AS1* as a protective factor for glioblastoma multiforme, as the patients with high expression of *ZNF197-AS1* have a longer lifespan than those with lower expression of *ZNF197-AS1* (10). In addition, *ZNF197-AS1* expression is also related to the clinicopathological features of breast cancer, including age, gender, stage, and T, M, and N stages (11). However, the possible involvement and predictive values of *ZNF197-AS1* in UM have yet to be characterized and thus become the focus of the current study. MicroRNAs (miRNAs or miRs) are a group of 21–23 nucleotides of single-stranded noncoding RNA and play an essential role in UM (12). MHENCR is a competitive endogenous RNA that specifically binds to *miR-425* and *miR-489*, upregulating the expression of their target genes *IGF1* and *SPIN1*. This action further activates the PI3K-Akt pathway, promoting the growth and metastasis of melanoma. Conversely, *miR-425* inhibits the progression of melanoma (13). miRNAs act as critical regulators of gene expression by binding to the 3′-untranslated region of mRNAs (14).

Previous studies have indicated that UM cells exhibit lower levels of autophagy (15). Autophagy, which regulates cellular homeostasis and survival through the degradation and recycling of intracellular components such as protein aggregates or organelles (16), represents a potential therapeutic avenue for cancer treatment (17). Therefore, it is necessary to manipulate the autophagy levels in UM cells to influence their progression and development. It has been reported that autophagy requires two ubiquitin-like protein conjugation systems, autophagy-related 5 (ATG5) and autophagy-related 8 (ATG8), with *GABARAPL1* being a member of the ATG8 family (18). *GABARAPL1* interacts with phospholipids and associates with intracellular membranes upon inhibiting lysosomal activity and accumulates within vesicles to regulate autophagy (19). Research has demonstrated that *GABARAPL1* can impact breast cancer development by regulating cellular autophagy (20). Moreover, *GABARAPL1* can modulate epithelial-mesenchymal transition in tumor cells by regulating autophagy (21). GABA(A)-receptor-associated protein like-1 (*GABARAPL1*), also known as autophagy-related 8 (ATG8) or glandular epithelial cell protein 1, belongs to the LC3/GABARAP protein family and plays significant roles in protein interaction and transportation, as well as autophagy, cell death, cell proliferation, and tumor progression (22, 23). *GABARAPL1* has been proposed as a potential prognostic biomarker for cutaneous melanoma (24).

Competitive endogenous RNA (ceRNA) networks based on RNA-RNA interactions represent a novel layer of gene regulation that controls both physiological and pathological processes (11, 25).

Based on the findings above and the evidence, this study aims to explore the potential molecular mechanisms underlying the involvement of lncRNA *ZNF197-AS1* in the growth and metastasis of UM. We downloaded the UM-related The Cancer Genome Atlas (TCGA) dataset from the UCSC Xena database and extracted differentially expressed lncRNAs, miRNAs, and mRNAs. Subsequently, we screened for the expression of the autophagy-related gene *GABARAPL1* and assessed its correlation with clinical-pathological features and prognosis data in patients with UM. Using Cytoscape, we constructed a ceRNA regulatory network consisting of *ZNF197-AS1*/*miR-425*/*GABARAPL1* and performed in vitro mechanistic validation using human regular retinal pigment epithelial cell line ARPE-19 and UM cell lines (92-1 and MP46). The proliferation, migration, and invasion abilities of UM cells were evaluated through the CCK-8 assay and Transwell experiment, whereas the levels of matrix metalloproteinase-2 (MMP-2) and MMP-9 in the supernatant of UM cells were measured using ELISA. Furthermore, we established a ceRNA network associated with lncRNA *ZNF197-AS1* to elucidate the interactions among the *ZNF197-AS1/miR-425/GABARAPL1* network in UM and to investigate the molecular mechanisms underlying the growth and metastasis of UM through a combination of bioinformatics analysis and in vitro experiments. Our study provides a novel understanding of the mechanisms involved in UM and offers new targets for its treatment.

## MATERIALS AND METHODS

### Data Source

The UM-related TCGA dataset, including RNA-Seq and miRNA-Seq, was downloaded from the UCSC Xena database. Meanwhile, the phenotypic and prognostic data of cancer tissue samples from 80 patients with UM were downloaded. UM patients with unknown clinicopathological characteristics were excluded, and 74 were finally used for characteristic clinicopathological analysis. All data types are FPKM data. Annotation information of datasets was obtained from the Gencode database. Finally, Perl language was applied for the ID conversion of the TCGA dataset (26).

### Survival Analysis

Using the Perl programming language, lncRNA and mRNA related to UM were extracted from the TCGA RNA-Seq dataset. Autophagy-related genes were obtained from the online platform (http://www.autophagy.lu/). The expression profiles of autophagy-related genes from the UM-associated TCGA dataset were extracted and merged with clinical prognosis data (OS) of patients with UM from the TCGA dataset. This integrated dataset of lncRNA, miRNA, and autophagy-related genes was used for survival analysis. Survival curves were constructed using the “survival” package in R (http://bioconductor.org/packages/survival/), and *P* values were calculated. A significance level of *P* < 0.05 was applied for interpretation.

### Construction of ceRNA Regulatory Network

Possible miRNAs binding to lncRNAs were predicted by the miRCode database and intersected with the prognosis-related miRNAs, resulting in the lncRNA_miRNA pair. Next, the screened miRNAs were used as candidate miRNAs. Then, potential mRNAs regulated by candidate miRNAs were predicted by the TargetScan database and subjected to intersection analysis with the prognosis-related mRNAs, yielding the miRNA_mRNA pair. Finally, the ceRNA regulatory network was visualized and mapped using the Cytoscape v3.6.0 software (27, 28).

### Correlation Analysis of *GABARAPL1* with the Clinicopathological Characteristics of UM Patients

The autophagy-related genes (ARG) *GABARAPL1* was divided into high- and low-expression groups based on the median value and integrated with clinicopathological characteristics. Clinical correlation analysis was performed using the R “limma” package and “pheatmap” package, and a heat map was drawn (29).

### Independent Prognostic Analysis of *GABARAPL1* with the Clinicopathological Characteristics of Patients with UM

*GABARAPL1* was integrated with the clinicopathological characteristics, survival time, and status of patients with UM and subjected to multivariate Cox regression analysis to determine whether *GABARAPL1* was an independent prognostic factor. Then, the R “survival” and “survminer” packages were used to draw forest maps according to the prognosis and calculate *P* values (30).

### Correlation Analysis of Immune Cells

The CIBERSORT algorithm was a deconvolution algorithm to characterize the cellular composition of complex tissues based on gene expression. The present study used the CIBERSORT algorithm to calculate the relative proportion of immune cells from the 80 patients with UM in the TCGA dataset. In addition, the correlation of *ZNF197-AS1*, *miR-425*, and *GABARAPL1* expression with immune cells was analyzed using the Correlation Coefficient (COR) test (31).

### Receiver Operating Characteristic Curves

To generate receiver operating characteristic (ROC) curves and calculate the area under curve (AUC) values for the prognostic marker gene *SPHK1*, we utilized the “survivalROC” package (https://CRAN.R-project.org/package=survivalROC). The “survivalROC” package was used to perform the ROC curve analysis, a widely used method for assessing the predictive accuracy of a biomarker in survival analysis. This analysis allows us to evaluate the discriminatory power of *SPHK1* as a prognostic marker for the studied condition. By plotting the ROC curve using the “survivalROC” package, we obtained a graphical representation of the relationship between the sensitivity and specificity of *SPHK1* in predicting patient outcomes. The AUC value, derived from the ROC curve, measures the overall performance of *SPHK1* as a prognostic marker, with a higher AUC indicating better predictive accuracy.

### Cell Culture and Treatment

Normal human retinal pigment epithelial cell line ARPE-19 and UM cell line 92-1 were obtained from Ningbo Mingzhou Biotechnology Co., Ltd. (Cat. No. B163956 and MZ-0410, Zhejiang, China) and cultured in DMEM medium (Cat. No. 12491015; ThermoFisher) containing 10% fetal bovine serum and 1% penicillin/streptomycin. UM cell line MP46 was purchased from ATCC (Cat. No. CRL-3298), and cultured in RPMI-1640 medium (Cat. No. 11875119; Gibco) supplemented with 20% fetal bovine serum (Cat. No. 10099141 C; Gibco) and 1% penicillin/streptomycin (Cat. No. 15140148; Gibco). All cell lines were maintained in a 37°C, 5% CO_2_ humidified incubator.

Using Lipofectamine 2000 reagent (11668019; ThermoFisher Scientific), MP46 and 92-1 cells were transfected with oe-NC + NC mimic plasmids, oe-*ZNF197-AS1* + NC mimic, and oe-*ZNF197-AS1* + *miR-425* mimic. The overexpression plasmid pCMV6-AC-GFP was purchased from Fenghui Biotechnology (Changsha, Hunan, China; FH1215), NC mimic from ThermoFisher Scientific (4464060), and *miR-425* mimic from Guangzhou RiboBio Co., Ltd. (Guangzhou, Guangdong, China; miR10003393-1-5). After 6 h, the medium was renewed, and cells continued to be cultured, collected, and used for subsequent experiments (32).

### RT-qPCR

Total RNA was extracted from cells using TRIzol reagent (Cat. No. 15596026; ThermoFisher Scientific), the concentration and purity of which were determined using a NanoDrop 2000 micro-UV spectrophotometer. Reverse transcription was performed using TaqMan MicroRNA Assays Reverse Transcription primer (Cat. No. 4427975; ThermoFisher Scientific) or PrimeScript RT reagent Kit (Cat. No. RR047A; Takara, Japan). RT-qPCR was conducted using the Applied Biosystems 7500 Fast Real-Time PCR System. U6 and GAPDH served as internal references, and the 2^–△△Ct^ method calculated the fold changes. TaKaRa synthesized the primer sequence, as shown in Table 1 (33).

**Table 1.** The primer sequence for RT-qPCR

Genes	Sequences (5′–3′)
ZNF197-AS1 (human)	Forward: 5′-AGGGATTTGGGGAATCCTCCT-3′ Reverse: 5′-GATGGCCATTGAGGTATGCAC-3′
miR-425 (human)	Forward: 5′-AATGACACGATCACTCCCGTTGA-3′ Reverse: Reverse universal primer
GABARAPL1 (human)	Forward: 5′-TCCCAGCTCTAGCGAAAAGC-3′ Reverse: 5′-CAAGTCCAGGTGCTCCCATC-3′
U6 (human)	Forward: 5′-GGCTCAGAATCACCCCATGT-3′ Reverse: Reverse universal primer
GAPDH (human)	Forward: 5′-AAAGCCTGCCGGTGACTAAC-3′ Reverse: 5′-TTCCCGTTCTCAGCCTTGAC-3′

### CCK-8 Assay

The CCK-8 kit (CA1210; Beijing Solarbio Science & Technology Co., Ltd., Beijing, China) was used to detect cell proliferation. First, cells were seeded in 96-well plates at 1 × 10^4^ cells/well density and cultured for 24 h, followed by transfection for 48 h. Next, the cells were incubated with 10 μL CCK-8 at 37°C for 3 h at 0, 24, 48, and 72 h of transfection. Finally, the absorbance of each well at 450 nm was measured on a microplate reader (34).

### Transwell Assay

First, 50 μL Matrigel was spread in the upper Transwell chamber and incubated for 2–3 h in an incubator. After detachment, the cells were made into cell suspension using a serum-free medium. Then 200 μL cell suspension was seeded into the upper Transwell chamber, and 800 μL medium containing 20% FBS was added into the lower chamber, followed by incubation at 37°C for 24 h. Next, the cells in the apical chamber were wiped with cotton, fixed with formaldehyde for 10 min, stained with 1% crystal violet, and allowed to stand at room temperature for 30 min. Finally, the cells were observed under a microscope and counted in at least four randomly selected microscope fields. Matrigel was not included in cell migration experiments, and the remaining steps were consistent with cell invasion experiments.

### ELISA

The supernatant of cells was harvested, where MMP-2 and MMP-9 were measured using ELISA kits (MMP-2 kit, JL22125-48T, Shanghai Jianglai Biotechnology Co., Ltd., Shanghai, China; MMP-9 kit, JL29650-48T, Jianglai Biotechnology) (35).

### Statistical Analysis

R (v4.1.1) software and SPSS 21.0 statistical software were used for statistical analysis. Measurement data were presented as means ± standard deviation. In addition, data obeying normal distribution and homogeneity of variance between two groups were compared using an unpaired *t*-test, and those among multiple groups were compared using one-way ANOVA with Tukey’s post hoc tests. A *P* value of <0.05 indicates a significant difference.

## RESULTS

### Bioinformatics Analysis Screens LncRNAs, miRNAs, and ARGs Associated with the Prognosis of Patients with UM

**Figure 1.**
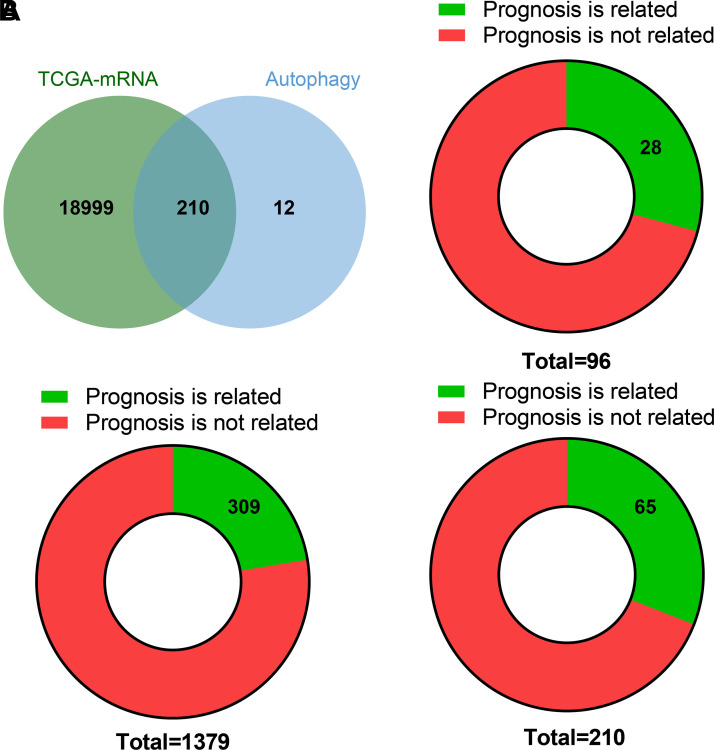
Screening of differentially expressed genes in the UM-associated lncRNA, miRNA, and mRNA expression datasets in the TCGA database. *A*: Venn diagram of mRNAs and ARGs. Green and blue represent mRNAs and ARGs, respectively, and the overlap represents ARGs in mRNAs. *B*: Pie charts represent the distribution of lncRNAs, miRNAs, and ARGs related to the prognosis of patients with UM. Green represents a correlation with the prognosis of patients with UM, and red represents no correlation with the prognosis of patients with UM. TCGA, The Cancer Genome Atlas; UM, uveal melanoma.

The ceRNA regulatory network may be a potential mechanism to influence the growth and metastasis of UM (36, 37). ARGs play an essential role in various cancers (38). However, there is little literature on ARGs for predicting the prognosis of patients with UM. Therefore, in this study, we obtained the UM-related lncRNAs, miRNAs, and mRNA expression datasets in TCGA from the UCSC Xena database and extracted 210 ARGs from the mRNA dataset (Fig. 1*A*). Next, these lncRNAs, miRNAs, and ARGs were subjected to Kaplan–Meier survival analysis. The results showed that 28 lncRNAs, 309 miRNAs, and 65 ARGs were closely related to the prognosis of patients with UM (Fig. 1*B*).

### The ceRNA Regulatory Network of *ZNF197-AS1*/*miR-425*/*GABARAPL1* May Be a Key Molecular Pathway Involved in UM Growth and Metastasis

**Figure 2.**
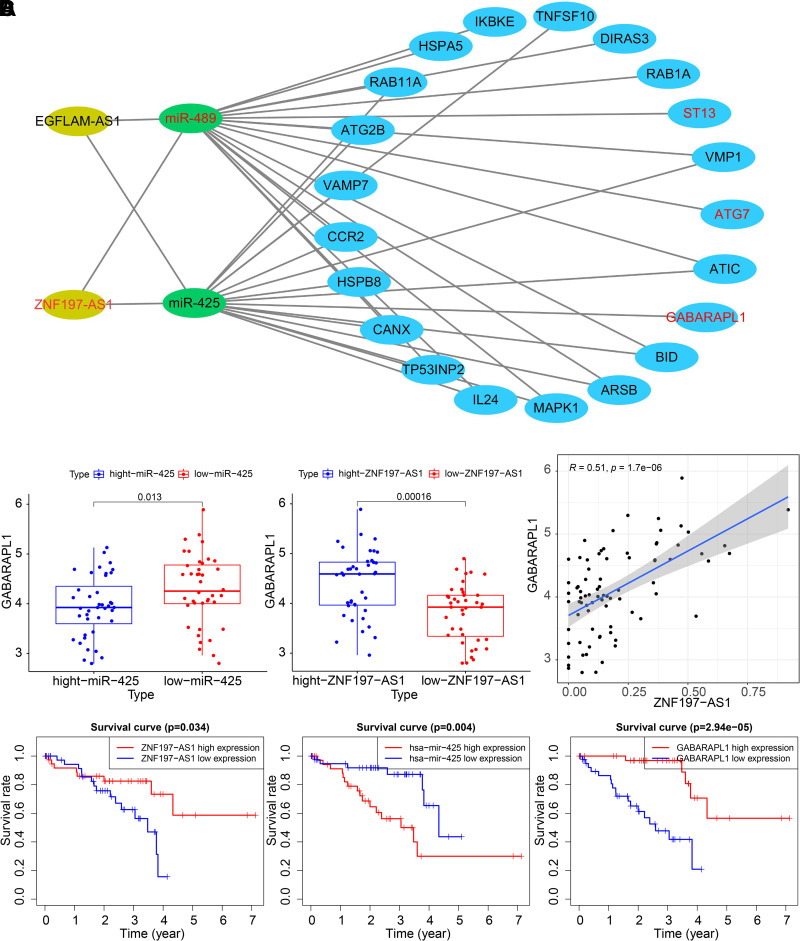
Construction of the ceRNA regulatory network of *ZNF197-AS1*/*miR-425*/*GABARAPL1* related to the prognosis of patients with UM. *A*: visualization results of the ceRNA regulatory network by the Cytoscape software. Yellow represents lncRNAs, green represents miRNAs, and blue represents mRNAs. The red font represents a positive correlation between the gene and the patient’s prognosis, and the black font represents a negative correlation between the gene and the patient’s prognosis. *B*: *GABARAPL1* expression in the *miR-425* and *ZNF197-AS1* high- and low-expression groups was based on their median expression values. *C*: the correlation analysis between *GABARAPL1* expression and *ZNF197-AS1* expression. *D*: survival analysis of patients with UM in the *ZNF197-AS1*, *GABARAPL1*, and *miR-425* high- and low-expression groups was based on their median expression values. The abscissa indicates the survival time, and the ordinate indicates the survival rate. Red lines indicate the high-expression group, and blue lines indicate the low-expression group. ceRNA, competing endogenous RNA; UM, uveal melanoma.

Next, to explore the ceRNA mechanism involved in regulating UM, we constructed a ceRNA regulatory network based on the lncRNA/miRNA/mRNA associated with the prognosis of patients with UM. First, the possible miRNAs binding to lncRNAs were predicted by the miRCode database, resulting in the lncRNA_miRNA pair. Then, potential mRNAs regulated by miRNAs were predicted by the TargetScan database, with miRNA_mRNA pair obtained. Finally, the ceRNA regulatory network was visualized by Cytoscape software (Fig. 2*A*). According to the ceRNA regulatory mechanism, lncRNAs promote the expression of downstream target genes via miRNA sponge. Only the *ZNF197-AS1*/*miR-425*/*GABARAPL1* signaling axis met the condition. As shown in Fig. 2, *B* and *C*, *GABARAPL1* expression was significantly decreased in the *miR-425* high-expression group but increased in the *ZNF197-AS1* high-expression group. In addition, *ZNF197-AS1* expression was positively correlated with *GABARAPL1* expression. The survival rate of UM patients with high *ZNF197-AS1* expression or *GABARAPL1* expression was much higher than that of UM patients with low *ZNF197-AS1* expression or *GABARAPL1* expression, whereas UM patients with high *miR-425* expression had a lower survival rate than those with low *miR-425* expression (Fig. 2*D*).

### The ARG *GABARAPL1* Is Closely Related to the Clinicopathological Characteristics of UM Patients

**Figure 3.**
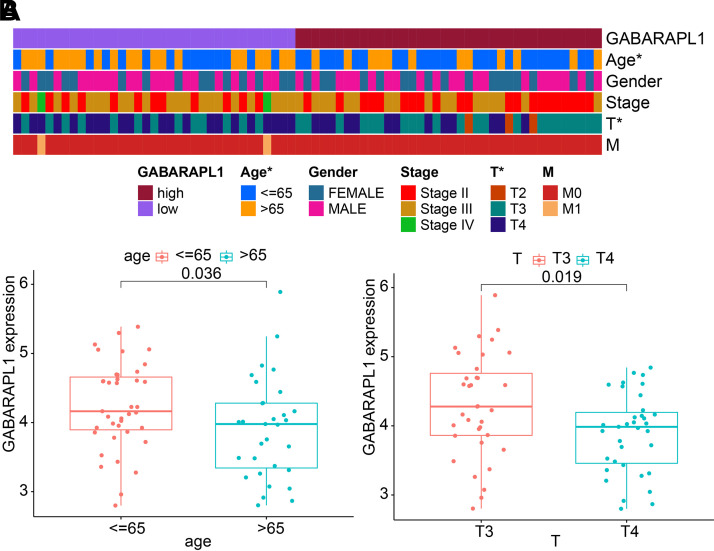
Correlation analysis of the ARG *GABARAPL1* with the clinicopathological characteristics of patients with UM. *A*: a heat map of the correlation of *GABARAPL1* with the clinicopathological characteristics of patients with UM. Below indicate the genes and clinical groups represented by each color block, among which *P < 0.05 corresponds to clinicopathological characteristics. *B*: *GABARAPL1* expression in different ages and T stages of patients with UM. There are 42 cases younger than 65, 32 cases over 65, 38 cases at T3, and 36 cases at T4. UM, uveal melanoma.

To investigate further the association between the autophagy-related gene *GABARAPL1* and the clinicopathological characteristics of patients with UM in the TCGA dataset, we divided the patients into two groups based on the median expression value of *GABARAPL1*. Patients with expression values higher than or equal to the median were classified as the *GABARAPL1* high-expression group, whereas those with values lower than the median were classified as the *GABARAPL1* low-expression group. The heatmap of clinical-pathological correlations (Fig. 3*A*) demonstrated a significant correlation between high and low expression of *GABARAPL1* and patient age and tumor growth in UM. There is a significant decrease in *GABARAPL1* expression in elderly patients with UM (Fig. 3*B*). Elderly patients often exhibit weaker immune systems and cellular autophagy functions, with *GABARAPL1* being a crucial protein in the autophagy process. The reduced expression level of *GABARAPL1* may indicate a decline in autophagy function in elderly patients. This weakening could lead to decreased clearance ability of tumor cells, promoting tumor growth and progression. Compared with T3 staging, there is a significant decrease in GABARAPL1 expression in T4 staging. The notable decrease in *GABARAPL1* expression in T4 staging suggests that as the tumor progresses, the expression of *GABARAPL1* may gradually diminish. This trend can serve as a marker of tumor degradation, aiding clinicians in evaluating the condition and devising treatment strategies.

### The ARG *GABARAPL1* Can Be Used as an Independent Prognostic Factor for UM

**Figure 4.**
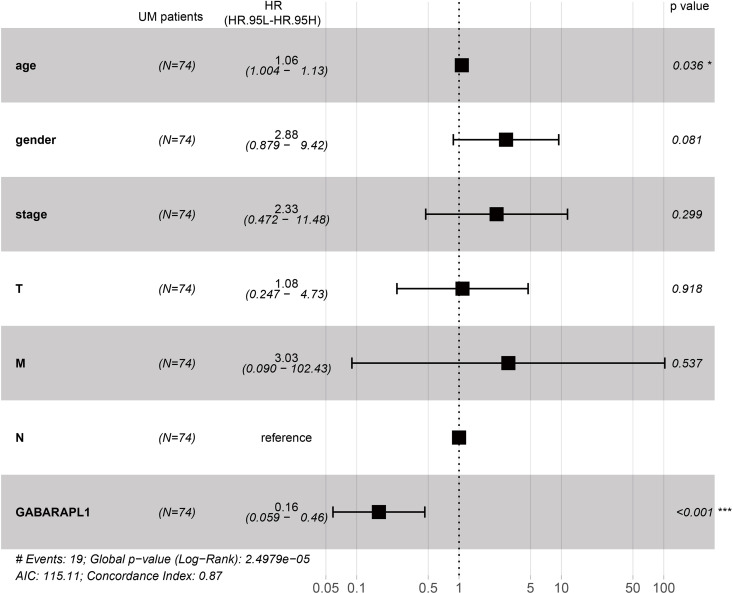
Independent prognostic analysis of the ARG *GABARAPL1* and the prognosis of patients with UM. A forest map of the multivariate Cox regression analysis. The *left* represents clinicopathological characteristics and gene name, *N* represents the case of patients with UM (74 cases), and the hazard ratio represents the risk rate, where a risk rate >1 indicates high risk and <1 indicates low risk. The *right* indicates the risk rate distribution of genes and *P* values, in which the *left* distribution indicates low risk and the *right* distribution represents high risk. UM, uveal melanoma.

The next focus of this study was to determine whether the ARG *GABARAPL1* can serve as an independent prognostic factor in patients with UM. Multivariate Cox regression analysis of the clinicopathological characteristics of UM patients with *GABARAPL1* expression in the TCGA dataset was performed. The results indicated the hazard ratio <1 of the *GABARAPL1* gene, a low-risk gene (Fig. 4). Thus, the ARG *GABARAPL1* can serve as an independent prognostic factor for patients with UM; the higher *GABARAPL1* expression reflected the higher survival rate of patients with UM.

### The ceRNA Regulatory Network *ZNF197-AS1*/*miR-425*/*GABARAPL1* May Inhibit UM Growth and Metastasis By Mediating Immune Pathways

**Figure 5.**
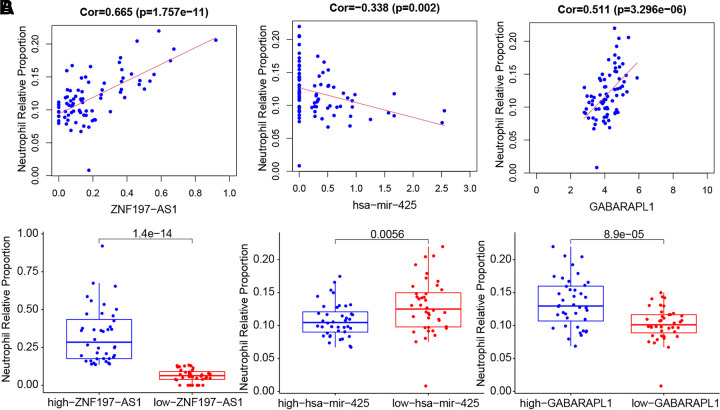
Correlation of the ceRNA regulatory network *ZNF197-AS1*/*miR-425*/*GABARAPL1* with immune cells in the peripheral blood samples of patients with UM in the TCGA dataset. *A*: neutrophil content in the *ZNF197-AS1*, *GABARAPL1*, and *miR-425* high- and low-expression groups was based on their median expression values. *B*: correlation between *ZNF197-AS1*, *miR-425*, and *GABARAPL1* expression and immune cells analyzed by the COR test. ceRNA, competing endogenous RNA; TCGA, The Cancer Genome Atlas; UM, uveal melanoma.

Previous research has shown that autophagy-related genes can influence the progression of malignant tumors through the immune pathway (35). To investigate the regulatory mechanism of the ceRNA regulatory network *ZNF197-AS1/miR-425/GABARAPL1* on the immune pathway in UM, we used CIBERSORT to calculate the relative proportions of immune cells in 80 patients with UM from the TCGA dataset. The correlation between the expression of *ZNF197-AS1/miR-425/GABARAPL1* and immune cells was analyzed using the COR test method. Our analysis revealed (Fig. 5, *A* and *B*) that the levels of neutrophils significantly increased in the high-expression groups of *ZNF197-AS1* and *GABARAPL1*, decreased in the high-expression group of *miR-425*, and that the expression of *ZNF197-AS1* and *GABARAPL1* was positively correlated with neutrophils, whereas the expression of *miR-425* had a slight negative correlation with neutrophil levels (correlation coefficient, −0.338).

Meanwhile, evidence has shown that neutrophils can kill cancer cells by releasing antimicrobial components, secreting different cytokines or chemokines, and interacting with other immune cells (39). Therefore, the ceRNA regulatory network *ZNF197-AS1*/*miR-425*/*GABARAPL1* may inhibit UM growth and metastasis by mediating the immune pathways.

### Correlation between ceRNA Regulatory Network *ZNF197-AS1*/*miR-425*/*GABARAPL1* and Prognostic Markers

**Figure 6.**
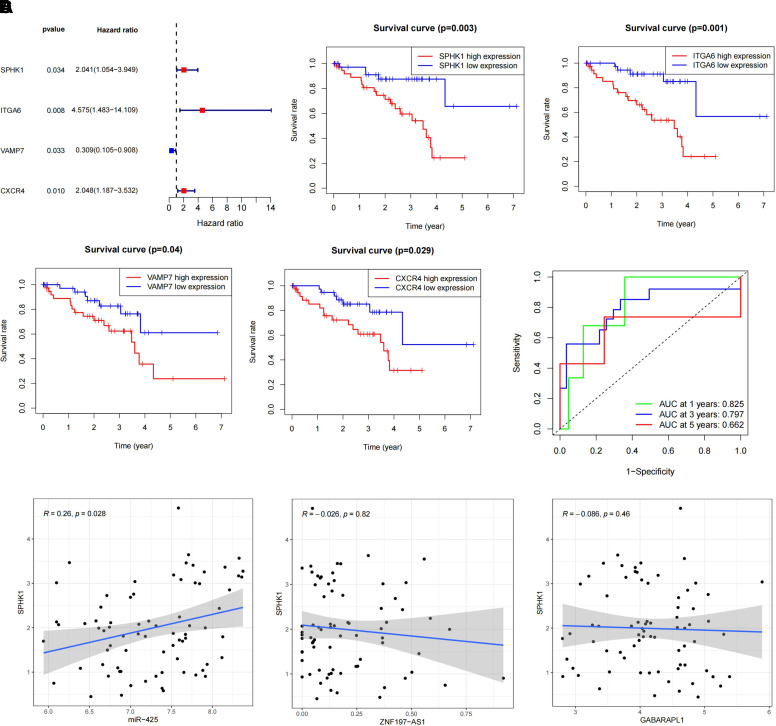
Correlation analysis between the ceRNA regulatory network and prognostic markers. *A*: independent survival analysis of prognostic genes, where red dots correspond to high-risk genes and blue dots correspond to low-risk genes. *B*: survival analysis of 80 patients with UM stratified into high- and low-expression groups based on the median expression of each gene. The *x*-axis represents survival time, and the *y*-axis represents the survival rate. The red line represents the high-expression group, whereas the blue line represents the low-expression group. *C*: ROC curve analysis of *SPHK1* for predicting the prognosis of patients with UM. The green, blue, and red curves represent the ROC curve analysis for 1, 3, and 5 years, respectively. *D*: correlation analysis of *ZNF197-AS1*/*miR-425*/*GABARAPL1* with the prognostic marker *SPHK1*. ceRNA, competing endogenous RNA; ROC curve, receiver operating characteristic curve; UM, uveal melanoma.

To identify prognostic markers, we performed independent survival analysis on genes associated with prognosis (Fig. 6*A*). We identified a total of four genes: *SPHK1, ITGA6, VAMP7,* and *CXCR4*. Among these, UM patients with high-expression levels of *SPHK1, ITGA6, VAMP7,* and *CXCR4* showed significantly lower survival rates compared with those with low-expression levels (Fig. 6*B*). Consequently, *VAMP7*, which was associated with low risk, was excluded from further analysis. We validated the accuracy of the independent prognostic genes in predicting the survival rates of patients with UM using ROC curves and found that only *SPHK1* accurately predicted patient prognosis (Fig. 6*C*). Based on these findings, we consider *SPHK1* to be a prognostic marker for patients with UM.

Furthermore, Spearman analysis was performed to investigate the correlation between the ceRNA regulatory network *ZNF197-AS1/miR-425/GABARAPL1* and the prognostic marker SPHK1, showing a positive trend between *miR-425* and *SPHK1*, consistent with the prognostic analysis results. However, *ZNF197-AS1* and *GABARAPL1* exhibited an opposite trend with SPHK1 in terms of prognosis, aligning with the ceRNA regulatory network trend but without significant correlation (Fig. 6*D*). We observed a significant positive correlation between *miR-425* and *SPHK1*, consistent with the results of the prognostic analysis. However, *ZNF197-AS1* and *GABARAPL1* showed an opposite trend in prognosis compared with *SPHK1*, which is in line with the ceRNA regulatory network trend but without significant correlation.

Our findings indicate that *SPHK1* can serve as a prognostic marker for patients with UM. In addition, the analysis revealed a significant positive correlation between *miR-425* and SPHK1, supporting the role of the ceRNA regulatory network in UM prognosis. However, no significant correlation was observed between *ZNF197-AS1*, *GABARAPL1*, and SPHK1.

### The ceRNA Regulatory Network *ZNF197-AS1*/*miR-425*/*GABARAPL1* Inhibits the Proliferation, Migration, and Invasion of UM Cells

**Figure 7.**
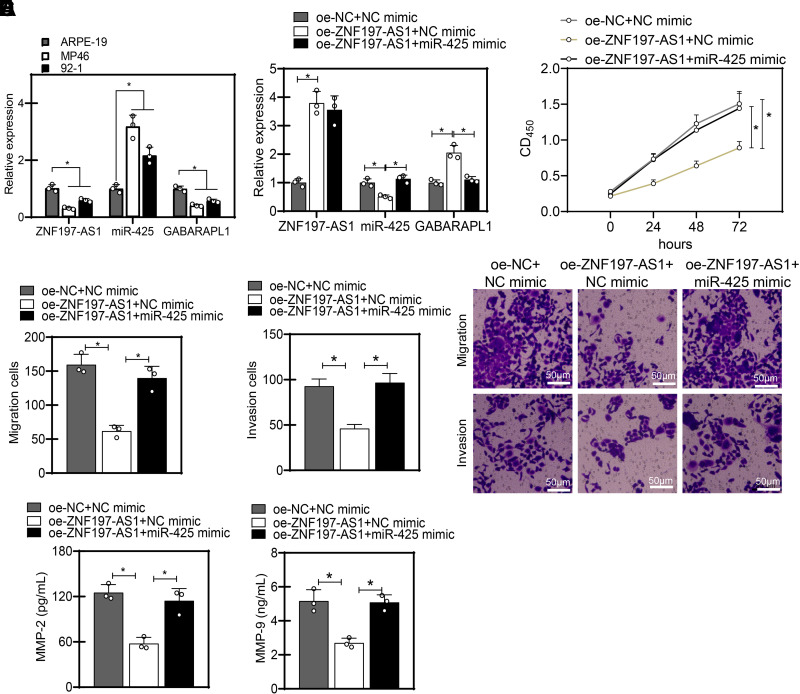
Inhibitory effects of the ceRNA regulatory network *ZNF197-AS1*/*miR-425*/*GABARAPL1* on the proliferation, migration, and invasion of UM MP46 cells. *A*: the expression of *ZNF197-AS1*, *GABARAPL1*, and *miR-425* in ARPE-19, MP46, and 92-1 cells was measured by RT-qPCR. *B*: the expression of *ZNF197-AS1*, *GABARAPL1*, and *miR-425* in MP46 cells was determined by RT-qPCR. MP46 cells were transfected with one-*ZNF197-AS1* or combined with *miR-425* mimic. *C*: the proliferation of MP46 cells was measured by CCK-8. *D*: the migration and invasion of MP46 cells were determined by Transwell assay (scale bar = 50 µm). *E*: MMP-2 and MMP-9 levels in the supernatant of MP46 cells were analyzed by ELISA. *P <0.05. Cell experiments were repeated three times. ceRNA, competing endogenous RNA; MMP, matrix metalloproteinase; UM, uveal melanoma.

To further investigate the impact of the ceRNA regulatory network *ZNF197-AS1*/*miR-425*/*GABARAPL1* on the malignant behavior of UM cells, we first examined the expression levels of *ZNF197-AS1*, *miR-425*, and *GABARAPL1* in human normal retinal pigment epithelial cells (ARPE-19) and UM cells (MP46 and 92-1) using RT-qPCR. Our experimental results demonstrated (Fig. 7*A*) that, compared with the control group of ARPE-19 cells, the UM cells in the experimental group exhibited significantly decreased expression of *ZNF197-AS1* and *GABARAPL1*, whereas the expression of *miR-425* was significantly increased. Moreover, the differences were more pronounced in MP46 cells than in 92-1 cells, leading us to select MP46 cells for subsequent functional research experiments.

**Figure 8.**
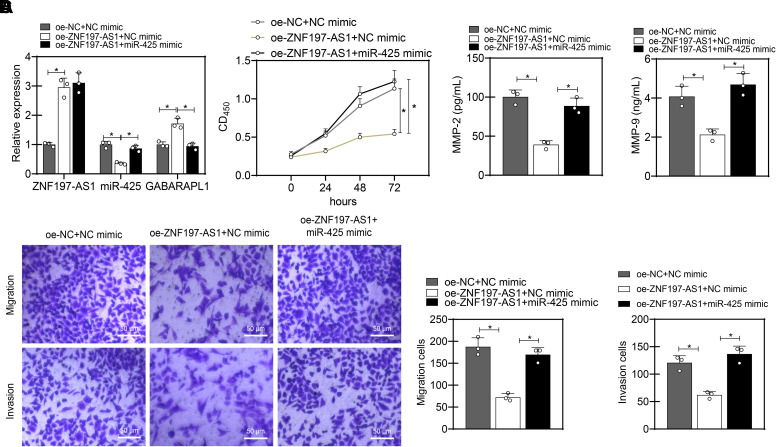
The impact of ceRNA regulatory network ZNF197-AS1/*miR-425*/GABARAPL1 on the proliferation, migration, and invasion capabilities of 92-1 cells. *A*: detection of ZNF197-AS1/*miR-425*/GABARAPL1 expression levels in 92-1 cells of each group by RT-qPCR. *B*: detection of proliferation capability of 92-1 cells in each group by CCK-8 assay. *C*: detection of migration and invasion capabilities of 92-1 cells in each group by Transwell assay (scale bar: 50 µm). *D*: detection of MMP-2 and MMP-9 levels in the supernatant of MP46 cells in each group by ELISA. *P < 0.05 compared between the two groups, and all cell experiments were repeated three times. ceRNA, competing endogenous RNA; MMP, matrix metalloproteinase.

The expression of *ZNF197-AS1* and *GABARAPL1* was upregulated, whereas that of *miR-425* was diminished in the MP46 and 92-1 cells overexpressing *ZNF197-AS1* (Fig. 7*B* and Fig. 8*A*). Conversely, upon additional *miR-425* overexpression, *miR-425* expression was elevated, but *GABARAPL1* expression was inhibited, in addition to unchanged *ZNF197-AS1* expression. In addition, the results of the CCK-8 and Transwell assay presented that ectopically expressed *ZNF197-AS1* suppressed the proliferation, migration, and invasion, whereas *miR-425* upregulation resulted in contrasting results (Fig. 7, *C* and *D*, and, Fig. 8, *B* and *C*). Furthermore, ELISA data presented decreased MMP-2 and MMP-9 levels in the supernatant of MP46 and 92-1 cells overexpressing *ZNF197-AS1,* whereas an opposite result was noted upon further *miR-425* overexpression (Fig. 7*E* and Fig. 8*D*). Collectively, the ceRNA regulatory network *ZNF197-AS1*/*miR-425*/*GABARAPL1* can impair the malignant phenotypes of UM cells.

## DISCUSSION

In this study, the combined application of bioinformatics analysis with the in vitro cell experiments demonstrated the mechanism underlying UM growth and metastasis. Specifically, *ZNF197-AS1* can competitively bind to *miR-425* and attenuate its binding to *GABARAPL1*, thus upregulating *GABARAPL1* expression and inhibiting UM growth and metastasis.

Initial data of this study suggested that *ZNF197-AS1* expression was associated with a good prognosis for patients with UM, as the survival rate of UM patients with high *ZNF197-AS1* expression was higher than that of UM patients with low *ZNF197-AS1* expression. *ZNF197-AS1* has been identified to be significantly associated with the prognosis of glioblastoma multiforme patients; patients with higher expression of *ZNF197-AS1* in the tumor tissues have a longer survival time compared with those with lower expression of *ZNF197-AS1* (10). The current study is the first to reveal the predictive value of *ZNF197-AS1* in the prognosis of patients with UM and requires further validation. Moreover, the correlation of *miR-425* expression with the clinicopathological characteristics of patients with UM warrants future verification, even though published references have identified that its high expression correlates with poor survival rates and acts as an independent prognostic factor in other malignancies (40–42).

The subsequent results of this study revealed that the ARG *GABARAPL1* was closely related to the clinicopathological characteristics of patients with UM and can serve as an independent prognostic factor for patients with UM, as the higher *GABARAPL1* expression reflected the higher survival rate of patients with UM. Importantly, the critical prognostic role of *GABARAPL1* in cutaneous melanoma has also been documented previously. Previous studies have indicated that *GABARAPL1* can accurately predict the prognostic outcomes of choroidal melanoma (37). Although choroidal melanoma cells exhibit a low level of autophagy (15), *GABARAPL1*, through its connection with phospholipids, inhibits lysosomal activity, binds to intracellular membranes, and accumulates in vesicles to regulate autophagy levels in tumor cells. Furthermore, research has shown that *GABARAPL1* can influence tumor angiogenesis by regulating the secretion of exosomes, thus impacting tumor growth, necrosis, and therapeutic efficacy.

Another important finding was that the ceRNA regulatory network *ZNF197-AS1*/*miR-425*/*GABARAPL1* played a vital role in the malignant phenotypes of UM cells, where *ZNF197-AS1* competitively bound to *miR-425* and then upregulated the expression of *GABARAPL1*, thus inhibiting the proliferation, migration, and invasion of UM cells. During the process of tumorigenesis, cancer cells not only evade the body’s regulatory mechanisms but also acquire the ability to influence local and systemic homeostasis. Specifically, tumors release cell factors, immunomediators, classic neurotransmitters, hypothalamic and pituitary hormones, biogenic amines, melatonin, and glucocorticoids, as confirmed in human and animal cancer models. This suggests that cancer can manipulate the central neuroendocrine and immune systems, at the expense of the host, to reset the body’s internal balance into a pattern conducive to its expansion. This mechanism elucidates the crucial role of the neuroendocrine system in cancer biology and provides new perspectives for future treatment strategies (43).

Many studies have confirmed that the regulatory ceRNA networks of lncRNAs, miRNAs, and mRNAs are associated with the pathogenesis of melanoma; the underlying mechanism is that lncRNAs act as miRNA sponges and thus reduce their regulatory effects on the target mRNAs (36, 44, 45). MMP-2 and MMP-9 have been extensively reported to promote tumor cell growth, migration, and invasion (46). This study demonstrated elevated levels of MMP-2 and MMP-9 in the supernatant of UM cells in the presence of *miR-425* upregulation, whereas opposite results were noted upon *ZNF197-AS1* overexpression, indicating the promoting effect of *miR-425* and the inhibiting effect of *ZNF197-AS1* on the proliferation, migration, and invasion of UM cells. However, the potential effect of *GABARAPL1* on the biological functions of UM cells remains to be elucidated to support current findings further.

Previous research indicates that melanin plays a crucial role in counteracting the detrimental effects of ultraviolet radiation and other environmental stressors. Although the presence of melanin can prevent the development of skin cancers, including melanoma, it may also be a necessary condition for the malignant transformation of melanocytes. Melanin is considered an effective antioxidant and sunscreen with radiation-protective and photoprotective properties; however, its photostability is poor, leading to mutagenic environments upon exposure to short-wavelength ultraviolet light. Melanin synthesis itself and its highly reactive intermediates exhibit cytotoxic, genotoxic, and mutagenic activities, stimulating the activation of glycolysis and hypoxia-inducible factor 1-alpha (HIF-1α), in addition to their immunosuppressive effects, which can contribute to melanoma progression and resistance to immunotherapy (47). Inducing melanin production pathways in cultured melanoma cells significantly upregulates HIF-1-dependent and independent pathways, indicating a critical role of melanin production in regulating cellular metabolism (48).

Besides, this study unveiled that the ceRNA regulatory network *ZNF197-AS1*/*miR-425*/*GABARAPL1* inhibited UM growth and metastasis by mediating immune pathways. Like numerous cancers, melanomas acquire multiple suppressive mechanisms, which usually act jointly, to escape innate and adaptive immune surveillance and destruction (49). Meanwhile, *miR-425* can affect the course of malignancies through immune pathways (42). Also, evidence has demonstrated the correlation of lncRNA functions in the occurrence and development of tumors, including melanomas, with the immune-related pathways (50, 51). The specific immune pathways regulated by *ZNF197-AS1*/*miR-425*/*GABARAPL1* warrant further experiments to identify the mechanism of this network in UM and develop potential therapies for UM.

This study also has certain limitations. In terms of the dataset, the sample size used for the analysis is relatively small, which may restrict the generalizability of our research findings. Regarding functional studies, our research primarily relies on in vitro experiments; therefore, further in vivo studies and clinical trials are needed to validate the therapeutic potential of ZNF197-AS1 in UM. In terms of mechanistic studies, this research validated the expression and functional relevance between *ZNF197-AS1/miR-425/GABARAPL1* using bioinformatics and wet experiments, but did not confirm the direct binding relationship between *ZNF197-AS1_miR-425/miR-425_GABARAPL1* using dual-luciferase assays, which is a limitation of this study. Based on the shortcomings identified, future research should focus on two aspects. First, in vivo experiments should be conducted to validate the *ZNF197-AS1/miR-425/GABARAPL1* axis and explore the therapeutic potential of targeting *ZNF197-AS1* in a clinical setting. Second, the role of *ZNF197-AS1* in other types of cancers should be investigated to determine whether the expression and functional characteristics observed in this study are universally applicable.

Currently, there are no reports on the biomarker role of *ZNF197-AS1* in UM, and further validation is necessary to establish its rationality as a biomarker for UM. However, many studies have shown that lncRNAs can serve as biomarkers for cancer clinical diagnosis and prognosis. LncRNA PCA3 is a highly expressed lncRNA in prostate tumors, and urine PCA3 testing is the first cancer detection method based on lncRNA that has been translated into clinical practice (52, 53). LncRNA HULC is a cancer-related lncRNA found in hepatocellular carcinoma patients, and it may serve as a potential noninvasive biomarker for cancer detection (54). Researchers found that RHPN1-AS1 is an oncoRNA in UM, which can serve as a candidate prognostic biomarker and a new therapeutic target for malignant UM (55). Based on the aforementioned research, there is substantial data support and confidence in considering ZNF197-AS1 as a biomarker. According to the results of this study, ZNF197-AS1 can be used for early diagnosis, prognosis prediction, and as a therapeutic target for UM, which may improve patient outcomes. It is speculated that high expression of ZNF197-AS1 is negatively correlated with poor prognosis in UM.

In conclusion, the present study identified UM-associated lncRNAs, miRNAs, and mRNAs and successfully constructed a ceRNA network, providing novel insights that may enhance the understanding of the functions of ceRNAs in UM, along with biomarkers for the development of therapies for UM. Of note, there was a lack of experimental validation in vivo. Nevertheless, the present results might be a foundation for establishing a mechanistic hypothesis for further experiments on clinical samples and animals.

## ETHICS APPROVAL AND CONSENT TO PARTICIPATE

This study did not involve human or animal material or data. Therefore, ethical approval was not required.

## DATA AVAILABILITY

All data supporting this study’s findings are available.

## DISCLOSURES

No conflicts of interest, financial or otherwise, are declared by the authors.

## AUTHOR CONTRIBUTIONS

C.Z. and S.W. conceived and designed research; performed experiments; analyzed data; interpreted results of experiments; prepared figures; drafted manuscript; edited and revised manuscript; approved final version of manuscript.
